# Nationwide Distribution of Dengue Virus Type 3 (DENV-3) Genotype I and Emergence of DENV-3 Genotype III during the 2019 Outbreak in Bangladesh

**DOI:** 10.3390/tropicalmed6020058

**Published:** 2021-04-21

**Authors:** Snigdha Rahman Titir, Shyamal Kumar Paul, Salma Ahmed, Nazia Haque, Syeda Anjuman Nasreen, Khondoker Shoaib Hossain, Fahim Uddin Ahmad, Sultana Shabnam Nila, Jobyda Khanam, Neaz Nowsher, Abu Mohammod Mayeenuddin Al Amin, Amdad Ullah Khan, Meiji Soe Aung, Nobumichi Kobayashi

**Affiliations:** 1 Department of Microbiology, Mymensingh Medical College, Mymensingh 2200, Bangladesh; titir.snigdha@gmail.com (S.R.T.); ahmed.salma51@yahoo.com (S.A.); nasreenm19@gmail.com (S.A.N.); nila1081@gmail.com (S.S.N.); dr.jkchaity1986@gmail.com (J.K.); 2 Netrokona Medical College, Netrokona 2400, Bangladesh; drshyamal10@yahoo.com; 3 Sheikh Hasina Medical College and Hospital, Jamalpur 1900, Bangladesh; drnaziahaque@gmail.com; 4 Department of Endocrinology, BIRDEM Academy, Dhaka 2302, Bangladesh; shoaib.mmc@gmail.com; 5 Department of Microbiology, TMSS Medical College, Bogura 5800, Bangladesh; fahim111mmc@gmail.com; 6 Department of Pathology, Khulna Medical College, Khulna 9100, Bangladesh; nneazrocky@gmail.com; 7 Department of Microbiology, Gazi Medical College, Khulna 9000, Bangladesh; mayeenuddinamin@gmail.com; 8 Department of Medicine, Mymensingh Medical College, Mymensingh 2200, Bangladesh; dramdadukhan@gmail.com; 9 Department of Hygiene, Sapporo Medical University School of Medicine, Sapporo 060-8556, Japan; meijisoeaung@sapmed.ac.jp

**Keywords:** dengue virus, type 3, genotype, IgM, Bangladesh

## Abstract

Bangladesh is an endemic region of dengue fever and experienced an unprecedented large outbreak with more than 100,000 confirmed cases in 2019. To understand the prevalence of dengue antibody in patients and molecular epidemiological characteristics of dengue virus (DENV) in this outbreak, a total of 179 blood samples were collected from patients in 10 districts (seven divisions) covering nearly the whole country from August to December 2019. DENV NS-1 was detected in 162 samples, among which DENV-specific IgM was positive in 119 samples (73.5%), including 60.5% samples also positive for DENV-specific IgG. Sequencing of the partial *C-prM* gene and its phylogenetic analysis revealed predominance of DENV type 3 genotype I, accounting for 93% of samples examined. DENV-3 genotype III was identified in two samples from separate districts, and only one DENV-2 cosmopolitan genotype was found in the capital city, Dhaka. These findings suggest the predominance of DENV-3 genotype I and occurrence of DENV-3 genotype III, associated with increased incidence of recent secondary infection in Bangladesh in 2019.

## 1. Introduction

Dengue virus (DENV) infection is a mosquito-borne, acute systemic disease and has become one of the major public health problems worldwide in the last few decades, with Asia having the greatest disease burden [1]. DENV is a member of the genus *Flavivirus* of the family *Flaviviridae*, and it contains four serotypes (DENV-1, -2, -3, and -4) which are further differentiated into genotypes. At present, the WHO Southeast Asia Region is considered hyperendemic for multiple DENV serotypes/genotypes [2]. Although a wide range of clinical manifestations represented by fever are seen in DENV infection in humans, secondary heterotypic DENV infection often results in severe disease previously known as dengue hemorrhagic fever (DHF) or dengue shock syndrome (DSS) [3], which is mediated by antibody-dependent enhancement of disease [4]. While the increased risk of such severe dengue disease is often related to a change in predominant DENV serotypes/genotypes, occurrence of DHF/DSS is commonly dependent on DENV types and their sequential order [3]. For example, DHF was associated with secondary infection with DENV-2 Asian genotype, but not with DENV-2 American genotype, after the initial epidemic of DENV-1 in Latin America [3,5,6]. This was also supported by the evidence that human sera infected with DENV-1 more efficiently cross-neutralized the DENV-2 American genotype than the Asian genotype [6]. Accordingly, for better control of dengue, it is important to understand its prevalence and monitor circulating DENV types in a population.

In Bangladesh, a large outbreak due to DENV-3 genotype II occurred in 2000 with more than 5000 hospitalized cases and continued until 2002 [2,7,8]. Thereafter, dengue was found at low frequency until 2016, with DENV-2 being recorded as the predominant causative virus, followed by DENV-1 [8,9,10]. Following the observation of re-emergence of DENV-3, as well as predominance of DENV-2 in 2017 [10,11,12], a large outbreak of dengue involving more than 10,000 cases occurred in Dhaka, the capital city of Bangladesh, in 2018. This outbreak was caused by multiple viruses including a dominant DENV-2, along with DENV-1 and DENV-3 [8,10,12,13]. A subsequent outbreak in 2019 caused a surge of dengue patients 10 times as high as that of previous year, i.e., 100,201 confirmed cases, among which half of the patients occurred in Dhaka, while the remaining cases were found across the rest of Bangladesh [14,15]. Although DENV-3 was described as predominant in the unprecedented outbreak in 2019 [8], genotypes of the responsible DENV are not yet clarified. The present study was conducted to understand prevalence of secondary dengue infections and reveal genetic characteristics of DENV causing the outbreak, in all areas of Bangladesh. The results indicated a high rate of dengue reinfection, with predominantly DENV-3 genotype I, as well as the emergence of DENV-3 genotype III.

## 2. Materials and Methods

**Figure 1:**
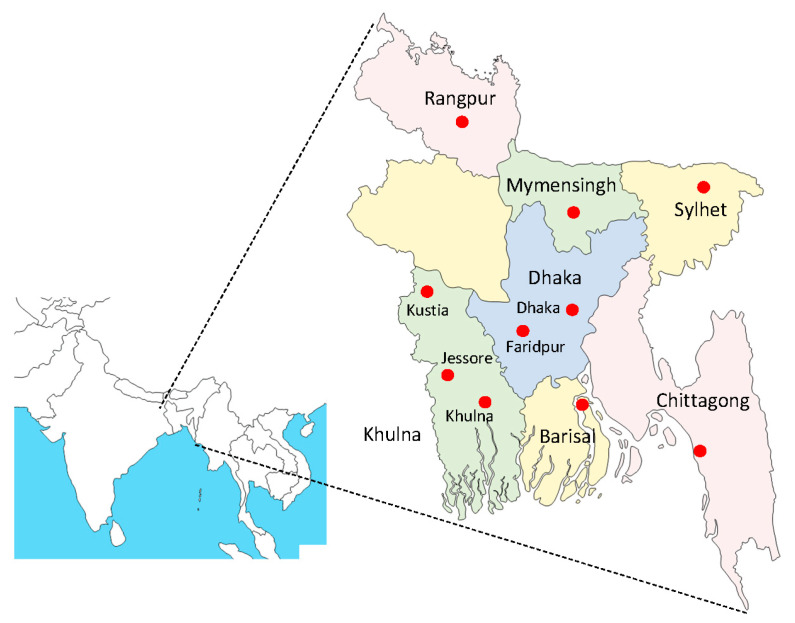
Map of cities in Bangladesh where blood samples were collected from dengue patients in this study. The eight divisions are shown in different colors.

Venous blood samples from febrile patients with clinically suspected dengue were collected from 10 districts in seven divisions in Bangladesh (Figure 1), from August to December 2019. In this year, the number of dengue cases increased from June and reached a peak in August, followed by rapid decrease [16]. Definition of suspected dengue cases included fever of ≥38 °C of less than 7 days duration and two or more symptoms or signs, as well as thrombocytopenia, as described previously [17]. Criteria of sample collection in this study were as follows: patients having fever over 38.5 °C and serum samples collected within 9 days post symptom onset. Written consent was obtained from individual patients.

**Figure 2:**
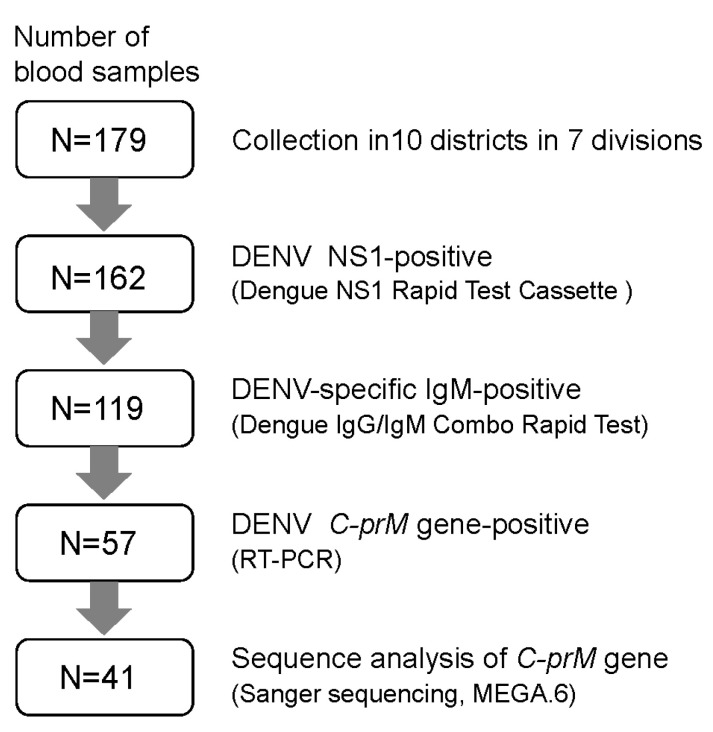
Flowchart representing the study of dengue virus and antibody with the use of blood samples in Bangladesh, 2019.

A flowchart of the analysis of dengue virus antigen, antibody, and gene is shown in Figure 2. Presence of DENV NS1 antigen was tested by the Dengue NS1 Rapid Test Cassette (Acro Biotech Inc., Rancho Cucamonga, CA, USA). Serum samples obtained from the blood samples via centrifugation were further analyzed for antibody and DENV gene.

DENV-specific IgM and IgG antibodies were detected by immunochromatographic test (ICT) using the Dengue IgG/IgM Combo Rapid Test (CTK Biotech, Inc., San Diego, CA, USA) according to the manufacturer’s instruction. The partial *C-prM* gene (511 bp) was amplified by RT-PCR using primer pairs as described previously [18]. Nucleotide sequences of amplified PCR products were determined by Sanger sequencing on an automated sequencer. Using partial *C-prM* gene sequences in the present study and those retrieved from the GenBank database, a phylogenetic dendrogram of the *C-prM* gene was constructed using the maximum-likelihood method with the MEGA6 software package (https://megasoftware.net/, accessed on 10 January 2021) [19]. The tree was statistically supported by bootstrapping with 1000 replicates, and genetic distances were calculated using the Kimura two-parameter model. The Clustal Omega program (https://www.ebi.ac.uk/Tools/msa/clustalo/, accessed on 12 January 2021) was also used for multiple alignment of nucleotide/amino-acid sequences and calculation of sequence identity. Partial *C-prM* gene sequences of representative 16 samples were deposited to GenBank database under accession Nos. MW599404–MW599419.

## 3. Results

A total of 179 blood samples were collected during the study period. Among the patients, males and females accounted for 61.5% and 38.5%, respectively, and the age range of 15 to 35 years included 48.4% of all the cases. Approximately 70% of patients were admitted to hospitals, while the remaining were outpatients. Among 179 samples, 162 (91%) were positive for the DENV NS1 antigen. Out of the 162 NS1-positive samples, DENV-specific IgM was detected in 119 samples (73.5%) by ICT. Both IgM and IgG were detected in 72 samples, accounting for 60.5% of IgM-positive samples (Table 1). IgM-positive samples were mostly derived in patients with a duration of fever of >3–5 days and >5–7 days (Figure S1, Supplementary Materials). Three samples were solely IgG-positive, while 40 samples were negative for both IgG and IgM. The DENV *C-prM* gene was detected in 57 samples. Although positive rates of IgM or *C-prM* were generally similar in all the study sites, divisions/districts with higher IgM-positive rates (>80%, e.g., Dhaka–Faridpur) showed relatively low RT-PCR-positive rates (<24%) (Table 1).

**Figure 3:**
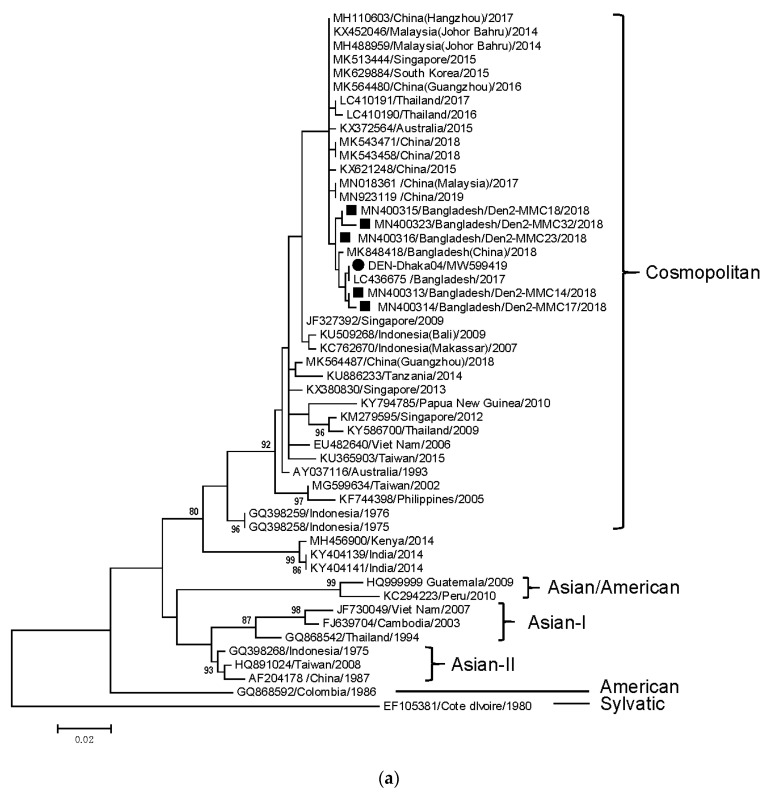

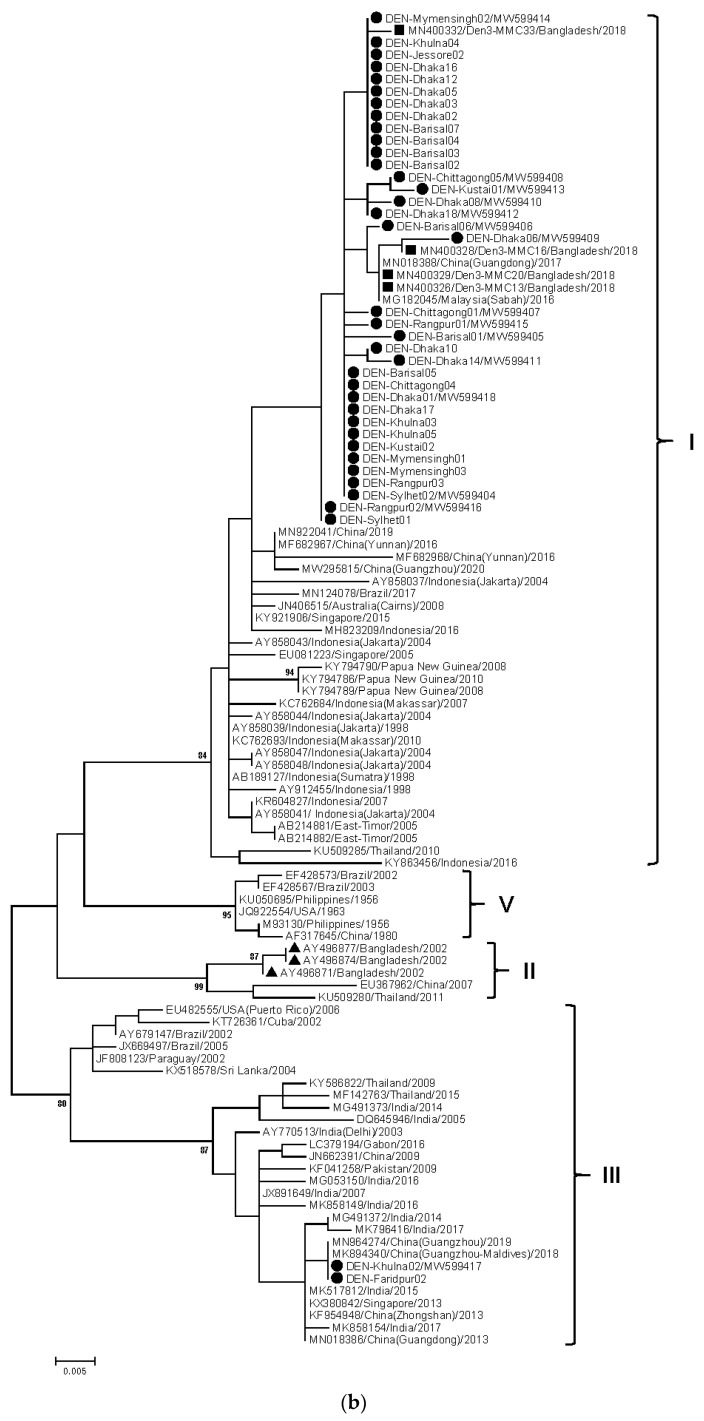
Phylogenetic dendrogram of partial *C-prM* gene of DENV-2 (**a**) and DENV-3 (**b**) in Bangladesh in 2019 outbreak and other diverse geographical locations, constructed using the maximum-likelihood method with the MEGA6 software package. Variation scale is described at the bottom. Percentage bootstrap support is indicated by the values at each node (values <80% were omitted). Closed circles and squares indicate DENV-2/DENV-3 in the present study (2019) and those reported previously (2018) in Dhaka [13], respectively. Triangles represent DENV-3 strains detected in Bangladesh in 2002. Genotypes of DENV-3/DENV-2 are shown on the right. Two DENV-3 samples were not included in dendrogram (**b**) because their nucleotide sequence length was considerably shorter than other sequences.

Although sequencing of partial *C-prM* gene was attempted for all 57 samples, accurate sequence data were obtained from 41 samples, after removal of samples showing low-quality data. Among the 41 samples, 40 samples belonged to DENV-3, while only one belonged to DENV-2. Phylogenetic analysis of *C-prM* genes revealed that most of DENV-3 belonged to genotype I, closely related to DENV-3 in Dhaka in 2018, as well as to recent strains in China and Malaysia (Figure 3b). Only two DENV-3 samples from Khulna and Faridpur (Dhaka division) were assigned to genotype III and clustered with strains in China, India, and Singapore detected after 2013. The partial *C-prM* gene sequences of genotype I DENV-3 exhibited >99% identity to each other, while they showed 93% identity to genotype III (data not shown). In the deduced C-prM amino-acid sequence, eight amino acids were distinct between genotype I and III (Figure S2, Supplementary Materials). Two cases of genotype III DENV-3 had identical nucleotide and amino acid sequences. Only one DENV-2 sample was detected in Dhaka, and it belonged to the Cosmopolitan genotype, being mostly close to DENV-2 in Dhaka, 2018 (Figure 3a).

## 4. Discussion

Expansion of the dengue outbreak in Bangladesh since 2017 is considered relevant to the introduction of DENV-3 into the region where preexisting DENV-1 and DENV-2 were circulating [11,12,13]. While having a low rate in 2017, DENV-3 showed a similar prevalence to DENV-2 in 2018 [13]. Our present study indicates that DENV-3 became predominant, displacing other DENV serotypes in 2019, when an unexpected large outbreak occurred in Bangladesh. Such a switching of major DENV serotype was described as involved in periodical outbreaks in endemic regions [20,21]. In western India, the dengue outbreak in 2017 was described as being associated with the replacement of dominant DENV-2 by emerging DENV-3 [22], which seems to be a similar situation to Bangladesh.

While DENV-3 re-emerged as a predominant virus after a 15 year period in Bangladesh, genotype I was predominant in 2018 and 2019, unlike genotype II that was prevalent in 2002 [23]. It is notable in the present study that two cases of DENV-3 with genotype III were identified, for the first time in Bangladesh. DENV-3 genotypes I and II have been prevalent in Asia, while genotype III is distributed across wider regions, i.e., Asia, the Caribbean, the Americas, and Europe [24]. Evidence from phylogenetic analysis suggests that genotype III DENV-3 in Bangladesh might have been brought in from neighboring countries, e.g., India or China. Furthermore, the two cases of genotype III DENV-3 had identical partial *C-prM* gene sequences, but they were detected in different districts, suggesting its potential spread within the country. Co-circulation of two DENV-3 genotypes I and III was reported in Colombia [25] and Malaysia [26]. Nevertheless, in Malaysia, an apparent shift from genotype III (2014–2015) to genotype I (2016–2017) was observed [26], which was different from the change in Bangladesh. Notably, genotype III viruses acquiring mutations were found to be associated with outbreaks in western India [22]. Although detailed genetic features in genotype III DENV-3 from Bangladesh remain to be determined, its prevalence should be carefully monitored, because switching of the main lineage even within the same serotype was found to be associated with outbreak [27]. Regarding DENV-2, cross-neutralization capability by antisera from individuals infected with DENV-1 was revealed to be different depending on genotypes [6]. Thus, it is also possible that the emerging genotype of DENV-3 may become dominant by selection with a preexisting antibody in the population. Nevertheless, it should be also noted that the introduction of new genotypes does not necessarily mean its growing prevalence in the population, because spread of new genotypes may depend on the transmissibility (from humans to mosquitoes, and vice versa), vector competence, and other environmental factors.

In the present study, DENV-specific IgM was detected in 73.5% of DENV-NS1-positive serum samples, among which 60% were positive for IgG. Because in this study, approximately 80% of blood samples were obtained within 7 days of febrile state, the concomitant detection of IgM and IgG may be suggestive of recent secondary infection on the basis of the timeline of dengue biomarkers [28]. The putative secondary infection rate was comparable to that described from global outbreak data (62%) [29]. Prevalence of IgG was generally similar among all the study sites including Dhaka, while previous major outbreaks appeared to take place mostly in Dhaka [9,11,12,13]. This may imply that dengue might have been potentially prevalent across the country. The relatively lower RT-PCR-positive rates associated with higher IgM-positive rates in some study sites suggest that DENV in the bloodstream was mostly neutralized by antibody and disposed of when blood samples were collected.

Secondary infection with DENV-2, DENV-3, and DENV-4, as well as primary infection with DENV-3, was shown to increase the risk of severe dengue disease [30]. In a recent study in northern Bangladesh, the rate of severe dengue was documented as 5.9% of patients, although the incidence of secondary infection was considerably lower than that of primary infection [31]. The predominance of DENV-3, emergence of its novel genotype III, and potentially high rate of secondary infection may pose a concern for the increased prevalence of dengue and its burden in Bangladesh. Hence, persistent molecular epidemiological surveillance of DENV may be valuable for the prevention and control of dengue outbreaks.

## Figures and Tables

**Table 1:** Detection of DENV-specific antibody and *C-prM* gene, as well as assignment of DENV serotype/genotype, in individual divisions/districts.

Division (District)	No. of NS1-Positive	IgM-Positive (%)		RT-PCR Positive (%)	Serotype (Genotype) *
		Total	IgM Only/IgM + IgG		
Barisal	22	16 (72.7)	4 (18.2)/12 (54.5)	10 (45.5)	DENV-3 (I)
Chittagong	18	14 (77.8)	7 (38.9)/7 (38.9)	7 (38.9)	DENV-3 (I)
Dhaka	40	27 (67.5)	7 (17.5)/20 (50)	18 (45)	DENV-2 (Cosmopolitan), DENV-3 (I)
Dhaka (Faridpur)	11	9 (81.8)	4 (36.4)/5 (45.5)	2 (18.2)	DENV-3 (III)
Khulna	16	9 (56.3)	5 (31.3)/4 (25)	6 (37.5)	DENV-3 (I, III)
Khulna (Jessore)	10	7 (70)	4 (40)/3 (30)	2 (20)	DENV-3 (I)
Khulna (Kustia)	12	9 (75)	4 (33.3)/5 (41.7)	4 (33.3)	DENV-3 (I)
Mymensingh	10	8 (80)	3 (30)/5 (50)	3 (30)	DENV-3 (I)
Rangpur	13	11 (84.6)	3 (23.1)/8 (61.5)	3 (23.1)	DENV-3 (I)
Sylhet	10	9 (90)	6 (60)/3 (30)	2 (20)	DENV-3 (I)
Total	162	119 (73.5)	47 (29.0)/72 (44.4)	57 (35.2)	

* Serotypes were assigned to 41 samples. Genotype was classified by phylogenetic analysis (Figure 3a,b).

## Supplementary Materials

The following are available online at https://www.mdpi.com/article/10.3390/tropicalmed6020058/s1: Figure S1. Detection of DENV-specific IgM and IgG by ICT and *C-prM* gene by RT-PCR in different periods (days) of fever; Figure S2. Alignment of partial C-prM amino-acid sequences of DENV-3 performed by Clustal Omega.
