# Supplementary material for: Nationwide Distribution of Dengue Virus Type 3 (DENV-3) Genotype I and Emergence of DENV-3 Genotype III during the 2019 Outbreak in Bangladesh
Source: Trop Med Infect Dis. 2021 Apr 21;6(2):58. doi: 10.3390/tropicalmed6020058 (PMC8167647; doi:10.3390/tropicalmed6020058)
Supplement: Supplementary file 1 [file tropicalmed-06-00058-s001.zip › Supplementary/Figure S1-tropicalmed6020058.pdf]

Supplementary Materials:

Nationwide Distribution of Dengue Virus Type 3 (DENV-3) Genotype I and Emergence of DENV-3 Genotype III during the 2019 Outbreak in Bangladesh

Snigdha Rahman Titir [1], Shyamal Kumar Paul [2], Salma Ahmed [1], Nazia Haque [3], Syeda Anjuman Nasreen [1], Khondoker Shoaib Hossain [4], Fahim Uddin Ahmad [5], Sultana Shabnam Nila [1], Jobyda Khanam [1], Neaz Nowsher [6], Abu Mohammod Mayeenuddin Al Amin [7], Amdad Ullah Khan [8], Meiji Soe Aung [9] and Nobumichi Kobayashi [9,*]

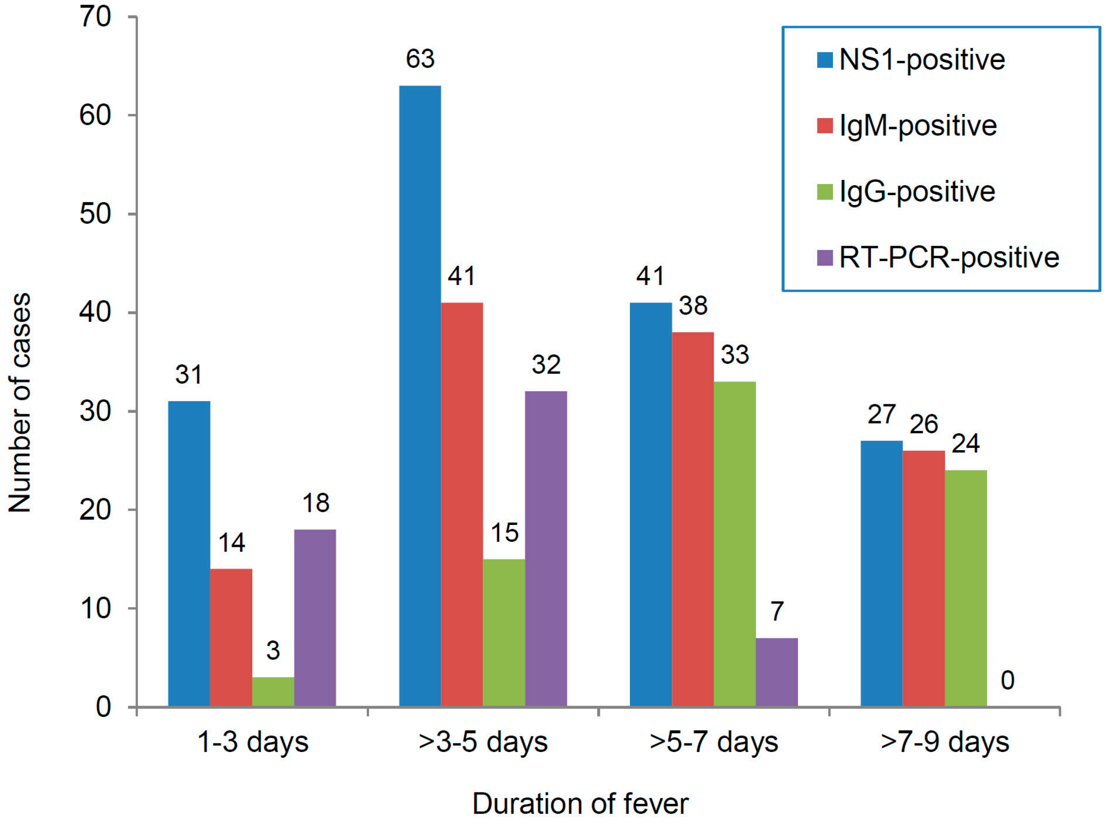

Figure S1. Incidence of dengue cases showing positive results of NS-1, IgM, IgG, and RT-PCR depending on duration of fever.
