# Supplementary material for: Nationwide Distribution of Dengue Virus Type 3 (DENV-3) Genotype I and Emergence of DENV-3 Genotype III during the 2019 Outbreak in Bangladesh
Source: Trop Med Infect Dis. 2021 Apr 21;6(2):58. doi: 10.3390/tropicalmed6020058 (PMC8167647; doi:10.3390/tropicalmed6020058)
Supplement: Supplementary file 1 [file tropicalmed-06-00058-s001.zip › Supplementary/Figure-S2-tropicalmed6020058.pdf]

```
DEN-Faridpur02      VSTGSQLAKRFSKGLLNGQGPMKLVMAFIAFLRFLAIPPTAGVLARWGTFKKSGAIKVLK  60
DEN-Khulna02        VSTGSQLAKRFSKGLLNGQGPMKLVMAFIAFLRFLAIPPTAGVLARWGTFKKSGAIKVLK  60
DEN-Mymensingh03    VSTGSQLAKRFSRGLLNGQGPMKLVMAFIAFLRFLAIPPTAGILARWGTFKKSGAIKVLR  60
DEN-Kustai01        VSTGSQLAKRFSRGLLNGQGPMKLVMAFIAFLRFLAIPPTAGILVRWGTFKKSGAIKVLR  60
DEN-Dhaka14         VSTGSQLAKRFSRGLLNGQGPMKLVMAFIAFLRFLAIPPTAGILARWGTFKKSGAIKVLR  60
DEN-Barisal06       -----QLAKRFSRGLLNGQGPMKLVMAFIAFLRFLAIPPTAGILARWGTFKKSGAIKVLR  55
DEN-Barisal01       VSTGSQLAKRFSRGLLNGQGPMKLVMAFIAFLRFLAIPPTAGILARWGTFKKSGAIKVLR  60
DEN-Barisal02       VSTGSQLAKRFSRGLLNGQGPMKLVMAFIAFLRFLAIPPTAGILARWGTFKKSGAIKVLR  60
DEN-Barisal03       VSTGSQLAKRFSRGLLNGQGPMKLVMAFIAFLRFLAIPPTAGILARWGTFKKSGAIKVLR  60
DEN-Barisal04       VSTGSQLAKRFSRGLLNGQGPMKLVMAFIAFLRFLAIPPTAGILARWGTFKKSGAIKVLR  60
DEN-Barisal05       VSTGSQLAKRFSRGLLNGQGPMKLVMAFIAFLRFLAIPPTAGILARWGTFKKSGAIKVLR  60
DEN-Barisal07       VSTGSQLAKRFSRGLLNGQGPMKLVMAFIAFLRFLAIPPTAGILARWGTFKKSGAIKVLR  60
DEN-Chittagong01    VSTGSQLAKRFSRGLLNGQGPMKLVMAFIAFLRFLAIPPTAGILARWGTFKKSGAIKVLR  60
DEN-Chittagong04    VSTGSQLAKRFSRGLLNGQGPMKLVMAFIAFLRFLAIPPTAGILARWGTFKKSGAIKVLR  60
DEN-Chittagong05    VSTGSQLAKRFSRGLLNGQGPMKLVMAFIAFLRFLAIPPTAGILARWGTFKKSGAIKVLR  60
DEN-Dhaka01         VSTGSQLAKRFSRGLLNGQGPMKLVMAFIAFLRFLAIPPTAGILARWGTFKKSGAIKVLR  60
DEN-Dhaka02         VSTGSQLAKRFSRGLLNGQGPMKLVMAFIAFLRFLAIPPTAGILARWGTFKKSGAIKVLR  60
DEN-Dhaka03         VSTGSQLAKRFSRGLLNGQGPMKLVMAFIAFLRFLAIPPTAGILARWGTFKKSGAIKVLR  60
DEN-Dhaka05         VSTGSQLAKRFSRGLLNGQGPMKLVMAFIAFLRFLAIPPTAGILARWGTFKKSGAIKVLR  60
DEN-Dhaka10         VSTGSQLAKRFSRGLLNGQGPMKLVMAFIAFLRFLAIPPTAGILARWGTFKKSGAIKVLR  60
DEN-Dhaka12         VSTGSQLAKRFSRGLLNGQGPMKLVMAFIAFLRFLAIPPTAGILARWGTFKKSGAIKVLR  60
DEN-Dhaka16         VSTGSQLAKRFSRGLLNGQGPMKLVMAFIAFLRFLAIPPTAGILARWGTFKKSGAIKVLR  60
DEN-Dhaka17         VSTGSQLAKRFSRGLLNGQGPMKLVMAFIAFLRFLAIPPTAGILARWGTFKKSGAIKVLR  60
DEN-Dhaka18         VSTGSQLAKRFSRGLLNGQGPMKLVMAFIAFLRFLAIPPTAGILARWGTFKKSGAIKVLR  60
DEN-Jessore02       VSTGSQLAKRFSRGLLNGQGPMKLVMAFIAFLRFLAIPPTAGILARWGTFKKSGAIKVLR  60
DEN-Khulna03        VSTGSQLAKRFSRGLLNGQGPMKLVMAFIAFLRFLAIPPTAGILARWGTFKKSGAIKVLR  60
DEN-Khulna04        VSTGSQLAKRFSRGLLNGQGPMKLVMAFIAFLRFLAIPPTAGILARWGTFKKSGAIKVLR  60
DEN-Khulna05        VSTGSQLAKRFSRGLLNGQGPMKLVMAFIAFLRFLAIPPTAGILARWGTFKKSGAIKVLR  60
DEN-Kustai02        VSTGSQLAKRFSRGLLNGQGPMKLVMAFIAFLRFLAIPPTAGILARWGTFKKSGAIKVLR  60
DEN-Mymensingh01    VSTGSQLAKRFSRGLLNGQGPMKLVMAFIAFLRFLAIPPTAGILARWGTFKKSGAIKVLR  60
DEN-Mymensingh02    VSTGSQLAKRFSRGLLNGQGPMKLVMAFIAFLRFLAIPPTAGILARWGTFKKSGAIKVLR  60
DEN-Rangpur01       VSTGSQLAKRFSRGLLNGQGPMKLVMAFIAFLRFLAIPPTAGILARWGTFKKSGAIKVLR  60
DEN-Rangpur02       VSTGSQLAKRFSRGLLNGQGPMKLVMAFIAFLRFLAIPPTAGILARWGTFKKSGAIKVLR  60
DEN-Rangpur03       VSTGSQLAKRFSRGLLNGQGPMKLVMAFIAFLRFLAIPPTAGILARWGTFKKSGAIKVLR  60
DEN-Sylhet01        VSTGSQLAKRFSRGLLNGQGPMKLVMAFIAFLRFLAIPPTAGILARWGTFKKSGAIKVLR  60
DEN-Sylhet02        VSTGSQLAKRFSRGLLNGQGPMKLVMAFIAFLRFLAIPPTAGILARWGTFKKSGAIKVLR  60
DEN-Dhaka06         -STGSQLGKRLSRGLLNGQGPMKLVMAFIAFLRFLAIPPTAGILVRWGTFKKSGAIKVLR  59
DEN-Dhaka08         VSTGSQLGKRLSRGLLNGQGPMKLVMAFIAFLRFLAIPPTAGILARWGTFKKSGAIKVLR  60
                         **.**:*:*****************************:*.**************:
```

```
DEN-Faridpur02       GFKKEISNMLSIINKRKKTSLCLMMILPAALAFHLTSRDGEPRMIVGKNERGKSLLFKTA  120
DEN-Khulna02         GFKKEISNMLSIINKRKKTSLCLMMILPAALAFHLTSRDGEPRMIVGKNERGKSLLFKTA  120
DEN-Mymensingh03     GFKREISNMLSIINRRKKTSLCLMMMLPATLAFHLTSRDGEPRMIVGKNERGKSLLFKTA  120
DEN-Kustai01         GFKREISNMLSIINRRKKTSLCLMMMLPATLAFHLTSRDGEPRMIVGKNERGKSLLFKTA  120
DEN-Dhaka14          GFKGEISNMLSIINRRKKTSLCLMMMLPATLAFHLTSRDGEPRMIVGKNERGKSLLFKTA  120
DEN-Barisal06        GFKREISNMLSIINRRKKTSLCLMMMLPATLAFHLTSRDGEPRMIVGKNERGKSLLFKTA  115
DEN-Barisal01        GFKREISNMLSIINRRKKTSLCLMMMLPATLAFHLTSRDGEPRMIVGKNERGKSLLFKTA  120
DEN-Barisal02        GFKREISNMLSIINRRKKTSLCLMMMLPATLAFHLTSRDGEPRMIVGKNERGKSLLFKTA  120
DEN-Barisal03        GFKREISNMLSIINRRKKTSLCLMMMLPATLAFHLTSRDGEPRMIVGKNERGKSLLFKTA  120
DEN-Barisal04        GFKREISNMLSIINRRKKTSLCLMMMLPATLAFHLTSRDGEPRMIVGKNERGKSLLFKTA  120
DEN-Barisal05        GFKREISNMLSIINRRKKTSLCLMMMLPATLAFHLTSRDGEPRMIVGKNERGKSLLFKTA  120
DEN-Barisal07        GFKREISNMLSIINRRKKTSLCLMMMLPATLAFHLTSRDGEPRMIVGKNERGKSLLFKTA  120
DEN-Chittagong01     GFKREISNMLSIINRRKKTSLCLMMMLPATLAFHLTSRDGEPRMIVGKNERGKSLLFKTA  120
DEN-Chittagong04     GFKREISNMLSIINRRKKTSLCLMMMLPATLAFHLTSRDGEPRMIVGKNERGKSLLFKTA  120
DEN-Chittagong05     GFKREISNMLSIINRRKKTSLCLMMMLPATLAFHLTSRDGEPRMIVGKNERGKSLLFKTA  120
DEN-Dhaka01          GFKREISNMLSIINRRKKTSLCLMMMLPATLAFHLTSRDGEPRMIVGKNERGKSLLFKTA  120
DEN-Dhaka02          GFKREISNMLSIINRRKKTSLCLMMMLPATLAFHLTSRDGEPRMIVGKNERGKSLLFKTA  120
DEN-Dhaka03          GFKREISNMLSIINRRKKTSLCLMMMLPATLAFHLTSRDGEPRMIVGKNERGKSLLFKTA  120
DEN-Dhaka05          GFKREISNMLSIINRRKKTSLCLMMMLPATLAFHLTSRDGEPRMIVGKNERGKSLLFKTA  120
DEN-Dhaka10          GFKREISNMLSIINRRKKTSLCLMMMLPATLAFHLTSRDGEPRMIVGKNERGKSLLFKTA  120
DEN-Dhaka12          GFKREISNMLSIINRRKKTSLCLMMMLPATLAFHLTSRDGEPRMIVGKNERGKSLLFKTA  120
DEN-Dhaka16          GFKREISNMLSIINRRKKTSLCLMMMLPATLAFHLTSRDGEPRMIVGKNERGKSLLFKTA  120
DEN-Dhaka17          GFKREISNMLSIINRRKKTSLCLMMMLPATLAFHLTSRDGEPRMIVGKNERGKSLLFKTA  120
DEN-Dhaka18          GFKREISNMLSIINRRKKTSLCLMMMLPATLAFHLTSRDGEPRMIVGKNERGKSLLFKTA  120
DEN-Jessore02        GFKREISNMLSIINRRKKTSLCLMMMLPATLAFHLTSRDGEPRMIVGKNERGKSLLFKTA  120
DEN-Khulna03         GFKREISNMLSIINRRKKTSLCLMMMLPATLAFHLTSRDGEPRMIVGKNERGKSLLFKTA  120
DEN-Khulna04         GFKREISNMLSIINRRKKTSLCLMMMLPATLAFHLTSRDGEPRMIVGKNERGKSLLFKTA  120
DEN-Khulna05         GFKREISNMLSIINRRKKTSLCLMMMLPATLAFHLTSRDGEPRMIVGKNERGKSLLFKTA  120
DEN-Kustai02         GFKREISNMLSIINRRKKTSLCLMMMLPATLAFHLTSRDGEPRMIVGKNERGKSLLFKTA  120
DEN-Mymensingh01     GFKREISNMLSIINRRKKTSLCLMMMLPATLAFHLTSRDGEPRMIVGKNERGKSLLFKTA  120
DEN-Mymensingh02     GFKREISNMLSIINRRKKTSLCLMMMLPATLAFHLTSRDGEPRMIVGKNERGKSLLFKTA  120
DEN-Rangpur01        GFKREISNMLSIINRRKKTSLCLMMMLPATLAFHLTSRDGEPRMIVGKNERGKSLLFKTA  120
DEN-Rangpur02        GFKREISNMLSIINRRKKTSLCLMMMLPATLAFHLTSRDGEPRMIVGKNERGKSLLFKTA  120
DEN-Rangpur03        GFKREISNMLSIINRRKKTSLCLMMMLPATLAFHLTSRDGEPRMIVGKNERGKSLLFKTA  120
DEN-Sylhet01         GFKREISNMLSIINRRKKTSLCLMMMLPATLAFHLTSRDGEPRMIVGKNERGKSLLFKTA  120
DEN-Sylhet02         GFKREISNMLSIINRRKKTSLCLMMMLPATLAFHLTSRDGEPRMIVGKNERGKSLLFKTA  120
DEN-Dhaka06          GFKREISNMLSIINRRKKTSLCLMMMLPATLAFHLTSRDGEPRMIVGKNERGKSLLFKTA  119
DEN-Dhaka08          GFKREISNMLSIINRRKKTSLCLMMMLPATLAFHLTSRDGEPRMIVGKNERGKSLLFKTA  120
                     *** **********:**********:***:******************************
```

```
DEN-Faridpur02      SGINMCTLIAMDLGEMCDDTVTYKCPHI-     148
DEN-Khulna02        SGINMCTLIAMDLGEMCDDTVTYKCPHI-     148
DEN-Mymensingh03    SGINMCTLIAMDLGEMCDDTVTYKCPVI-     148
DEN-Kustai01        SGINMCTLIAMDLGEMCDDTVTYKCPLI-     148
DEN-Dhaka14         SGINMCTLIAMDLGEMCDDTVTYKCPLI-     148
DEN-Barisal06       SGINMCTLIAMDLGEMCDDTVTYKCPLIT     144
DEN-Barisal01       SGINMCTLIAMDLGEMCDDTVTYKCPLI-     148
DEN-Barisal02       SGINMCTLIAMDLGEMCDDTVTYKCPLIT     149
DEN-Barisal03       SGINMCTLIAMDLGEMCDDTVTYKCPLIT     149
DEN-Barisal04       SGINMCTLIAMDLGEMCDDTVTYKCPLIT     149
DEN-Barisal05       SGINMCTLIAMDLGEMCDDTVTYKCPLIT     149
DEN-Barisal07       SGINMCTLIAMDLGEMCDDTVTYKCPLIT     149
DEN-Chittagong01    SGINMCTLIAMDLGEMCDDTVTYKCPLI-     148
DEN-Chittagong04    SGINMCTLIAMDLGEMCDDTVTYKCPLI-     148
DEN-Chittagong05    SGINMCTLIAMDLGEMCDDTVTYKCPLI-     148
DEN-Dhaka01         SGINMCTLIAMDLGEMCDDTVTYKCPLIT     149
DEN-Dhaka02         SGINMCTLIAMDLGEMCDDTVTYKCPLI-     148
DEN-Dhaka03         SGINMCTLIAMDLGEMCDDTVTYKCPLI-     148
DEN-Dhaka05         SGINMCTLIAMDLGEMCDDTVTYKCPLI-     148
DEN-Dhaka10         SGINMCTLIAMDLGEMCDDTVTYKCPLI-     148
DEN-Dhaka12         SGINMCTLIAMDLGEMCDDTVTYKCPLI-     148
DEN-Dhaka16         SGINMCTLIAMDLGEMCDDTVTYKCPLI-     148
DEN-Dhaka17         SGINMCTLIAMDLGEMCDDTVTYKCPLI-     148
DEN-Dhaka18         SGINMCTLIAMDLGEMCDDTVTYKCPLI-     148
DEN-Jessore02       SGINMCTLIAMDLGEMCDDTVTYKCPLI-     148
DEN-Khulna03        SGINMCTLIAMDLGEMCDDTVTYKCPLI-     148
DEN-Khulna04        SGINMCTLIAMDLGEMCDDTVTYKCPLI-     148
DEN-Khulna05        SGINMCTLIAMDLGEMCDDTVTYKCPLI-     148
DEN-Kustai02        SGINMCTLIAMDLGEMCDDTVTYKCPLI-     148
DEN-Mymensingh01    SGINMCTLIAMDLGEMCDDTVTYKCPLI-     148
DEN-Mymensingh02    SGINMCTLIAMDLGEMCDDTVTYKCPLI-     148
DEN-Rangpur01       SGINMCTLIAMDLGEMCDDTVTYKCPLI-     148
DEN-Rangpur02       SGINMCTLIAMDLGEMCDDTVTYKCPLI-     148
DEN-Rangpur03       SGINMCTLIAMDLGEMCDDTVTYKCPLI-     148
DEN-Sylhet01        SGINMCTLIAMDLGEMCDDTVTYKCPLI-     148
DEN-Sylhet02        SGINMCTLIAMDLGEMCDDTVTYKCPLI-     148
DEN-Dhaka06         SGINMCTLIAMDLGEMCDDTVTYKCPLI-     147
DEN-Dhaka08         SGINMCTLIAMDLGEMCDDTVTYKCPLI-     148
                    ************************** *
```

**Fig. S2** Alignment of partial CprM amino acid sequences of DENV-3 performed by Clustal Omega. Asterisk at the bottom indicates consensus amino acid. Amino acids of genotype III (Faridpur02, Khulna02) on top that are distinct from those of genotype I (all other viruses) are shown in red.
